# Can Transgenic Maize Affect Soil Microbial Communities?

**DOI:** 10.1371/journal.pcbi.0020128

**Published:** 2006-09-29

**Authors:** Christian Mulder, Marja Wouterse, Markus Raubuch, Willem Roelofs, Michiel Rutgers

**Affiliations:** 1 Laboratory for Ecological Risk Assessment, National Institute for Public Health and the Environment, Bilthoven, Netherlands; 2 Department of Soil Biology and Plant Nutrition, University of Kassel, Witzenhausen, Germany; 3 Central Science Laboratory, Sand Hutton, York, United Kingdom; University of Wageningen, Netherlands

## Abstract

The aim of the experiment was to determine if temporal variations of belowground activity reflect the influence of the Cry1Ab protein from transgenic maize on soil bacteria and, hence, on a regulatory change of the microbial community (ability to metabolize sources belonging to different chemical guilds) and/or a change in numerical abundance of their cells. Litter placement is known for its strong influence on the soil decomposer communities. The effects of the addition of crop residues on respiration and catabolic activities of the bacterial community were examined in microcosm experiments. Four cultivars of Zea mays L. of two different isolines (each one including the conventional crop and its Bacillus thuringiensis cultivar) and one control of bulk soil were included in the experimental design. The growth models suggest a dichotomy between soils amended with either conventional or transgenic maize residues. The Cry1Ab protein appeared to influence the composition of the microbial community. The highly enhanced soil respiration observed during the first 72 h after the addition of *Bt*-maize residues can be interpreted as being related to the presence of the transgenic crop residues. This result was confirmed by agar plate counting, as the averages of the colony-forming units of soils in conventional treatments were about one-third of those treated with transgenic straw. Furthermore, the addition of *Bt*-maize appeared to induce increased microbial consumption of carbohydrates in BIOLOG EcoPlates. Three weeks after the addition of maize residues to the soils, no differences between the consumption rate of specific chemical guilds by bacteria in soils amended with transgenic maize and bacteria in soils amended with conventional maize were detectable. Reaped crop residues, comparable to post-harvest maize straw (a common practice in current agriculture), rapidly influence the soil bacterial cells at a functional level. Overall, these data support the existence of short *Bt*-induced ecological shifts in the microbial communities of croplands' soils.

## Synopsis

There is a huge number of studies regarding the effects of exposure to transgenic maize pollen on nontarget insects. These concerns are due to the key role of pollinating insects in ecosystem functioning. Many results are highly debated and controversial. However, soil microbes as nontarget organisms are neglected. This is surprising, given the wide variation in agro-ecosystems that arises from several bacterial-driven processes (soil respiration, priming effect of organic matter, litter quality and quantity, and many others). Should the inclusion of bacteria in these nontarget organisms be regarded as artifactual? This seems unambiguous, as soil bacteria themselves are known to be the most crucial element of ecosystem functioning. Effects on the metabolism of soil bacteria grown in microcosms treated with either transgenic or conventional maize straw were studied in the laboratory. In contrast to the authors' expectations, empirical evidence is available to show different utilization of carbon compounds, especially of carbohydrates, according to the concentration of the *Bt* toxin. To their knowledge, this is the first concrete attempt to address the belowground impact of transgenic maize under controlled situations. The next step must be the assessment of the extent to which bacteria could be endowed with transgenic plant DNA in the soil.

## Introduction

**Figure 1:**
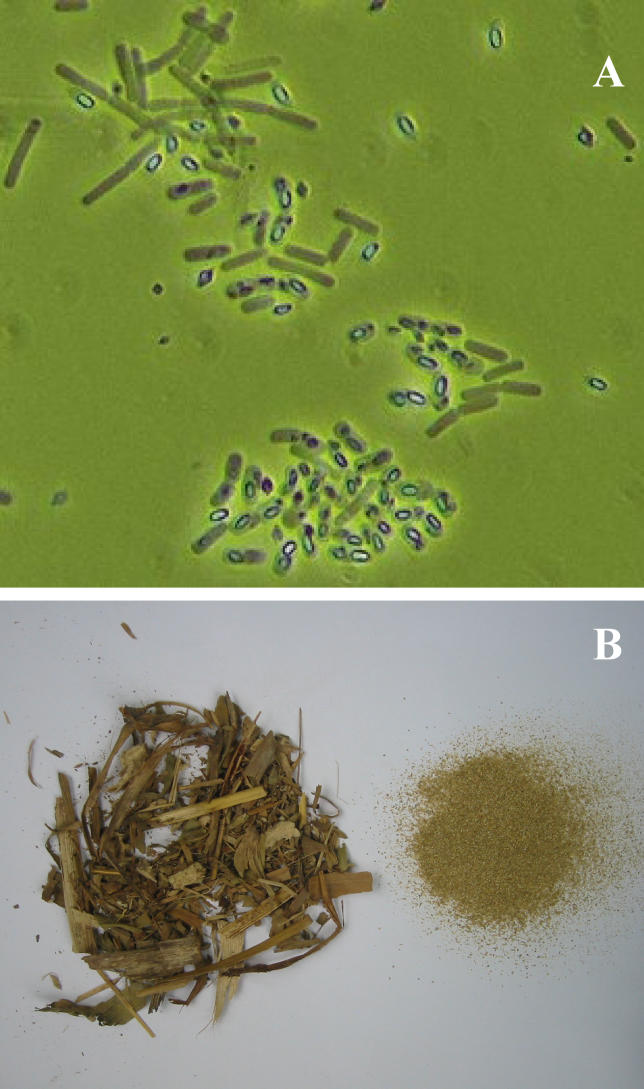
*Bt* Parasporal Crystals in Maize and One Amended Soil (A) Photo of B. thuringiensis (ssp. kurstaki) with protein crystals, hyaline spores, and vegetative cells (elongate, opaque cells) by courtesy of Dr. Bjarne Munk Hansen, Danmarks Miljøundersøgelser. (B) Transgenic crop residues and soil sample after the addition of reaped maize straw shortly before starting the experiment (complete straw analysis available as Table S1).

Bacillus thuringiensis is a gram-positive spore-forming bacterium which produced parasporal crystals during sporulation that are pathogenic to insect and some other organisms [1–8, Figure 1A]. In contrast to lepidopterans feeding on crystals alone, a clear pattern of synergism was demonstrated for lepidopterans feeding on the strongly pathogenic mixture of spores and crystals [9,10]. Preparations of bacterial spores and crystalline proteins are widely used as *Bt* insecticides for the control of insect pests of crops. *Bt*-toxins are classified based on their specific activity against invertebrates [11–14]. Cry1Ab is a toxin commonly used against the European corn borer (Ostrinia nubilalis Hübner) and can be produced either as a full-length protoxin, as by Monsanto, or as the Novartis-produced, truncated, preactivated toxin. Besides reports of insects that seem to have acquired resistance to larvicidal toxins belonging to the Cry1 protein family [e.g., 15], nontarget effects of the toxin produced by the insecticidal *cry1Ab* gene released in the root exudates of transgenic B. thuringiensis corn are still unclear. The intrinsic heterogeneity of the rhizosphere of, among others, single maize roots, is unique and well-investigated [16,17], in contrast to the bulk soil. Yet, significant nontarget effects outside the rhizosphere have not been detected [e.g., 18–21]. Microbial communities occurring belowground under genetically engineered *Bt* crops are in fact less investigated, and possible effects on soil microbes remain a concern [20,21]. The toxins may accumulate in soils after post-harvest maize straw is ploughed in (Figure 1B), and, subsequently, toxins bind on soil components such as clay minerals [22] and humic acids [23]. However, to our knowledge, to what extent this kind of environmental disturbance is recognizable in the bacterial communities has not been addressed yet. Our aim is to get a better ecological insight into the microbial community using the metabolic fingerprints of bulk soil bacteria under controlled conditions.

## Results/Discussion

**Figure 2:**
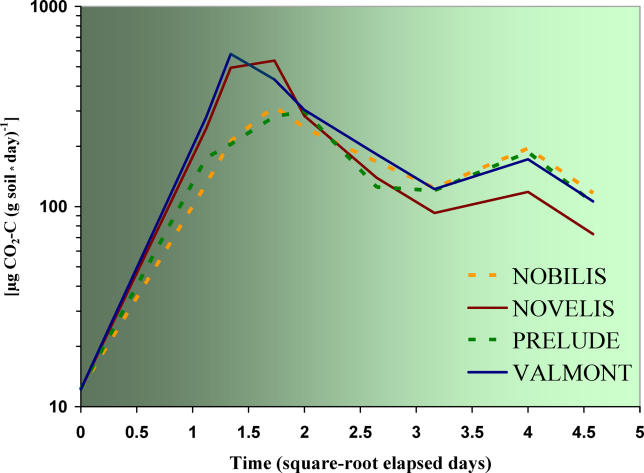
Amount of CO_2_-C after Addition of Maize Residues Temporal variance in gross soil respiration of a stagnic luvisol after the addition of maize straw. Dotted lines for crop residues from conventional maize, continuous lines for transgenic maize. Untreated soils without any addition of straw showed initial respiration values of 12 μg CO_2_-C at d 0 (*n* = 5). MON810 (Novelis) and Event 176 (Valmont) are transgenic. The differences between these cultivars and their mother plants were significant: conventional straw did not enhance soil respiration as much as transgenic straw (ANOVA: *p* < 0.00001).

Differences occurred in the microbial respiration activity of soil samples with added transgenic straw in comparison with soil samples with conventional maize residues (Table 1, Figure 2). The short-term increase in microbial respiration activity was shown by a peak in CO_2_ production between the 24th and the 72nd hour of all soil samples treated with transgenic maize residues (either Novelis or Valmont). The soil control remained, on average, at 5.5 ± 3.2 μg CO_2_-C g^−1^ d^−1^ during the entire experiment. These rates of CO_2_ production suggest different mineralization patterns of *Bt*-maize straw in comparison with conventional maize straw (*p* = 0.0004). However, all rates of CO_2_ production were roughly comparable after four days (Figure 2). Addition of easily degradable substrates, such as straw, always enhance soil respiration, but here a much higher activity was clearly correlated with transgenic plant material. Soils amended with *Bt*-maize straw had, 30 h after the addition of the transgenic plant material, 73% more respiration than soils amended with conventional maize straw. After 43 h, it increased to 157%. However, after 72 h, this positive trend changed, as soil respiration decreased rapidly to 61%. During the initial phase of the experiment (Figure 2), the 20 amended replicates showed a positive relationship between higher concentrations of Cry protein in the added straw and the rates of CO_2_ production: the first isoline (Nobilis | Novelis) showed *p* < 0.00001, and the second isoline (Prelude | Valmont) showed *p* = 0.0008. The latter exhibit much higher standard deviations (SD), Prelude with ±39 and transgenic Valmont with ±69 SD.

**Table 1:**
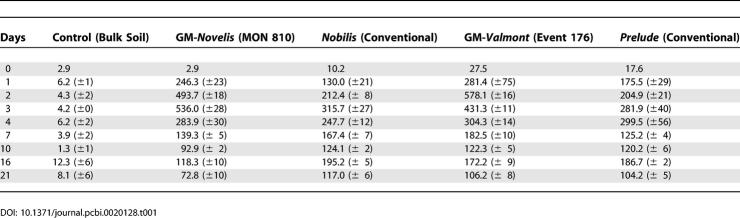
Daily Respiration of Soils Amended with Straw from Different Cultivars of Maize (μg CO_2_/g Dry Soil ± SD) Whose Cultivars Show Strong Differences (ANOVA: *p* < 0.0002)

### Different Carbon Sources

**Figure 3:**
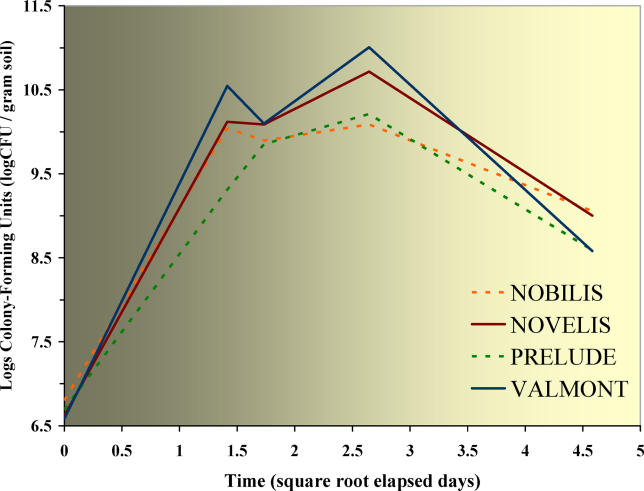
Plate Counts in Time CFU growing curves in amended soils. Dotted lines for crop residues from conventional maize, continuous lines for transgenic maize. Transgenic maize straws show a much higher number of CFUs than conventional straws during the first week (ANOVA: *p* = 0.0296), but the differences between all four maize cultivars are not significant (ANOVA: *p* = 0.1389).

Parallel with measurements of soil respiration, both the counting of colony-forming units (CFUs) and the measurement of metabolic fingerprints by BIOLOG were performed along a time gradient. At the beginning of the experiment, no significant difference was detectable between the 5 × 2 soil microcosms (average CFU equals 5 × 10^6^ with *p* = 0.088). The addition of maize resulted in a pulse of viable cells 30 h after the soil amendment, exactly as previously detected by soil respiration (Figure 2), but this pulse was broken 43 h after the addition of crop residues (Figure 3). The conventional Nobilis, which started with the highest number of CFUs (6.34 × 10^6^ at the beginning of the experiment) but depicted on average the lowest number of CFUs (1.06 × 10^10^) after the addition of straw, was the only exception. The curves of CFUs grown after the addition of transgenic material show a second, much sharper peak 7 d after the amendment (Figure 3). The plate count showed that the agar-grown colonies in the soils amended with straw from the two transgenic cultivars, Novelis and Valmont, were, respectively, 2.78× and 3.77× more than in the soils amended with Nobilis straw. After the addition of crop residues, the average CFU (2.19 ± 3.39 × 10^10^) remained four orders of magnitude higher than the average CFU before the start of the experiment (4.99 ± 1.21 × 10^6^). For both isolines, the averages of agar-grown CFUs did not show significant difference after 21 d (*p* > 0.86). Straw from Valmont, the transgenic cultivar with the lowest concentration of the Cry1Ab protein and the highest content of proteins, fats, sugars, and starch (Table S1), showed the highest number of CFUs. Seventy-six percent of the CFUs grew during the first 48 h, but without significant differences between transgenic and conventional crops.

The utilization of 31 sole-carbon sources by bacterial communities of soils in the presence of increasing concentrations of the Cry1Ab protein was measured by a color development assay performed on BIOLOG microtitre plates (see Materials and Methods). Two kinds of transgenic maize residues were taken into account and were compared with conventional crop residues and straw-free EcoPlates. Metabolic reactions were measured by the change of the dye from colorless to purple (individual well color development (WCD)), indicating bacterial heterotrophic growth on a specific substrate as a carbon and energy source. When individual carbon sources were compared according to treatment, there were differences with many of these substrates over time during the entire experiment. The rates of color change over time in each well of the EcoPlate showed that 12 of 31 substrates seemed to be more specific for the bacterial communities in soils amended with transgenic straw (Table 2 and Table S2, respectively). Two-thirds of these *Bt*-sensitive substrates were carbohydrates (Table S2). Most of them (α-D-lactose, β-methyl-D-glucoside, D-cellobiose, D-mannitol, D-xylose, Glucose-1-phosphate, and N-acetyl-D-glucosamine) were positively correlated with the presence of the Cry1Ab protein (Table S2), whereas i-erythritol was the only carbohydrate negatively correlated with the Cry1Ab protein (Table 2). Another carbohydrate present in this 31 sole-carbon source set, D,l-α-glycerol phosphate, did not show any significant response to the measured concentration of the Cry1Ab protein (Table S2).

**Table 2:**
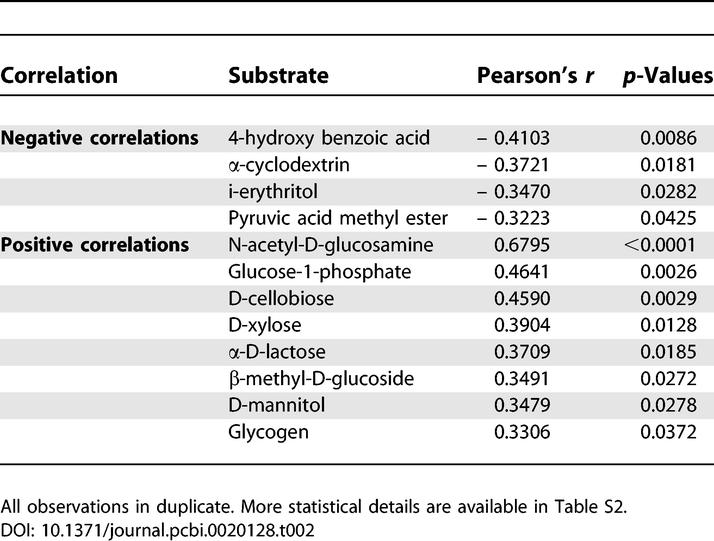
Correlations between WCD Values of BIOLOG Carbon Sources and the Concentration of the Cry1Ab Protein as Measured at the Beginning of the Experiment

Regardless of the elapsed days, the counts of a given color development (a WCD value that is specific for the respective carbon source) showed differences within each isoline in the catabolic activity of the soil bacteria during the entire experiment. Such differences in the potential carbon utilization are clearly related to the addition of transgenic material and describe a lower utilization of some substrates. Among the 31 × (31 – 1) = 930 possible matrix correlations between the EcoPlate substrates, 9.3% correlations were (slightly) responsive at α = 0.10 and 30.7% were highly significant at α = 0.05. There was no prevalence of either direct or indirect correlations between these 31 independent carbon sources, as 52% of the correlations were positive and 48% were negative.

**Figure 4:**
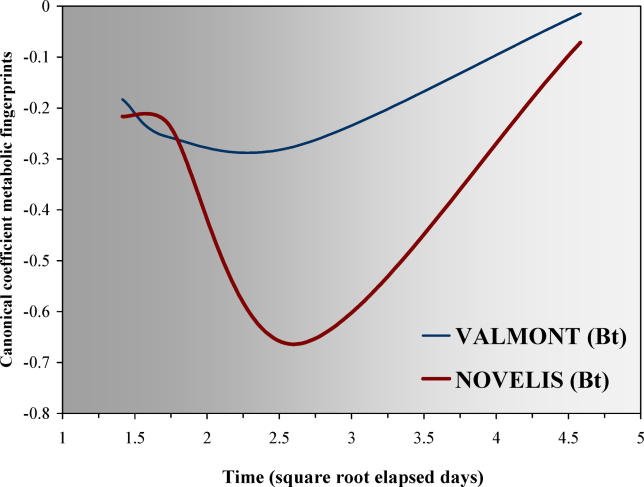
Multivariate Analysis of the Soils Amended with Transgenic Maize PRCs of the summarized metabolic fingerprints of the two *Bt*-maize straws (both Nobilis and Prelude as conventional isoline control level as horizontal *x*-axis) according to all BIOLOG EcoPlate carbon sources (*n* = 3 in duplicate). Day 3 is the most significant by time for all maize cultivars (SD = ± 0.242, *p* = 0.064, *F*-ratio = 2.66).

Time was also modeled as canonical covariable (see [24] and Materials and Methods). Figure 4, a principal response curves (PRC) diagram as defined in Van den Brink and Ter Braak [24], showed that the reaction patterns over time of the microbial community under *Bt*-maize straw with higher concentrations of the Cry1Ab protein are entirely different from the communities treated with other transgenic crop residues or those treated with conventional maize straw (*p* = 0.0020). However, the BIOLOG responses seemed to be opposite to those of the plate counts, as here the transgenic cultivar Novelis, whose straw showed the highest concentration of the Cry1Ab protein and the lowest content of proteins, fats, sugars, and starch (Table S1), exhibited the highest reaction pattern over time. According to [24], the PRCs for conventional maize seemed unrelated to other response patterns due to time and *Bt*-treatment.

Using the untreated control as an appropriate nesting specification (see Materials and Methods) to fit the data for initial soil respiration to a multilevel generalized linear model, β-methyl-D-glucoside (F_1,38_ = 5.15) was the only carbohydrate that showed a significant (inverse) correlation between treatment with straw containing a high concentration of the Cry1Ab protein and carbon utilization (Table 3), although the aforementioned carbohydrate group as a complete chemical guild depicts a direct correlation between treatment with transgenic straw and carbon utilization. An interesting point of discussion is that almost all these carbohydrates do not seem to support most culturable bacteria in experimental grasslands. In research on bulk soil bacteria growing in the Swiss BIODEPTH study area [25], 50% of the 31 available carbon sources showed a highly significant error probability of the linear regression slope being different from zero. Moreover, these authors suggested an increased overall catabolic activity in their nutrient-rich Lolium perenne grasslands [25]. In our case, 39% of the available carbon sources show a highly significant correlation of a different-from-zero slope with the Cry1Ab protein (Table 2). Robust differences in the potential carbon utilization were also consistent with previous field studies on nutrient-poor upland grasslands. For instance, some carbon substrates negatively correlated with the concentration of the Cry1Ab protein, such as the neutral amino acid L-phenylalanine (Table S2), were compounds described as typical for many unimproved grasslands of the British Isles [26].

**Table 3:**
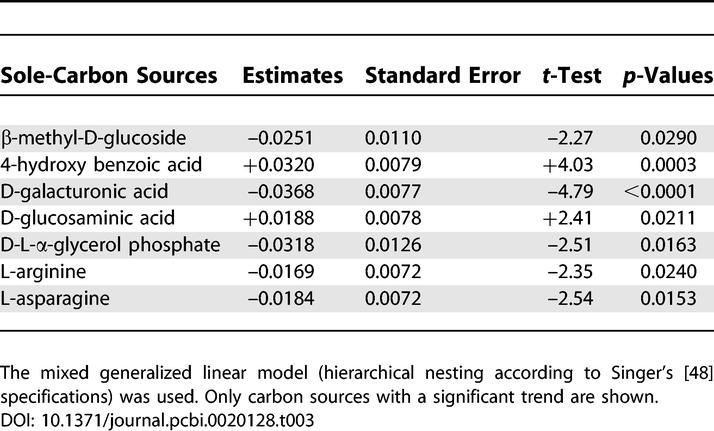
Catabolic Activity of Soil Bacteria (as Measured by the Change of the Dye from Colorless to Purple) on the BIOLOG Carbon Sources (Time as Fixed Effect, and Log-Transformed WCD Values as 31 Dependent Variables)

We may conclude that the possibly adaptive radiation of bulk soil bacteria in our microcosms shortly after the addition of *Bt*-maize straw was much more easily detectable in the laboratory than in the field (a mosaic of blocks under experimental conditions with trampling and mowing [25], or a gradient of upland grasslands with much greater utilization of sugars in nutrient-rich sites, possibly reflecting substrate availability [26]). Actually, pulsing microbial effects of transgenic maize straw became more evident due to the high sugar content of some added *Bt*-material (1.11% dry weight in the transgenic cv. Valmont, twice the content of its mother plant Prelude). Even some amino acids exhibited a significant correlation between the concentration of the Cry1Ab protein and the WCD, namely L-asparagine (F_1,38_ = 6.46) and L-arginine (F_1,38_ = 5.53). Also, these amino acids are considered to be important for the compositional analysis of maize [27]. It is unclear whether the different estimates of catabolic activity as WCD after addition of transgenic material are real or are stochastic results. These differences in time among the log-transformed WCD estimates are often small; in the field, where the errors are larger, the same temporal uncertainties apply to other possible nesting specifications.

These possibilities need to be addressed in future analyses. Overall, however, the trend was a clearly different catabolic activity of the bacteria in all the microcosms treated with transgenic plant material in comparison with those of the control and of the soils amended with conventional crop residues.

### Conclusions

In microcosm experiments we examined the effects of the addition of maize straw on properties such as respiration and catabolic activities of the bacterial community. We hypothesized that variations in the belowground activity would reflect the influence of transgenic plants, due to the effect of the Cry1Ab protein on the bulk soil bacteria, and hence on the ability of the microbial community to metabolize carbon sources. We combined microbiological techniques and mixed generalized linear models to facilitate a more comprehensive understanding of possible stress.

The performed growth models established a striking dichotomy between the soils amended with either conventional or transgenic straw. The concentrations of the Cry1Ab protein clearly influenced the composition of the microbial community. Evidence for increasing soil respiration during the second and third days of the treatment can be interpreted in relation to the presence of transgenic plant material.

Microbial uptake of sugars seemed to be affected by the presence of transgenic material (*p* = 0.0015). We did not detect any correlation between the increased microbial activity below a transgenic straw layer and its content of starch, lignin, hemicellulose, and cellulose. The addition of straw from *Bt*-cultivars pointed also to an increased microbial consumption of BIOLOG carbohydrates (*p* < 0.0001).

Our data suggest that, in contrast to previous studies, the introduction of transgenic maize influences abundance, diversity, and ecosystem functioning of the bulk soil bacteria. Overall, the data provide support for the notion that a *Bt*-induced adaptive radiation may occur rapidly in the microbial communities below maize fields.

There were short but robust differences in the soil microbial community after the addition of transgenic plant material. *Bt*-maize straw may cause not only an increase of the abundance of CFUs, but can also select for particular ecophysiological traits. This may account for the similarity in long-term response of soils treated with different maize residues. Therefore, the extracted microbial communities from these microcosms remain rather comparable to those of spatially homogeneous soils.

These results may have important implications for the ecological risk-assessment of genetically modified organisms at the community level [21,28]. Bacteria are abundant and very diverse [29–33] and can respond rapidly to environmental perturbations [32–36], for instance during competition for nutrients required for bacterial growth and activity [34–36]. All the results in these studies showed an evident response of bacteria in bulk soil to the addition of transgenic plant residues.

The technique of metabolic fingerprinting enabled the detection of rapid shifts in the functional diversity of the microbial community after the introduction of the Cry1Ab toxin in the environment. A resulting question for further research is therefore to determine genetically which components of the microbial community contribute to the increase of activity shortly after the treatment with transgenic material.

## Materials and Methods

### Soil and plant materials

The soil was a stagnic luvisol containing 75% silt, 21% clay, and 4% sand from the Hebenshausen 37249 site (Meierbreite, 51°21′ N, 9°52′ E) of the Department of Soil Ecology and Plant Nutrition at the University of Kassel (Germany). Total soil carbon and total soil nitrogen were analyzed by dry combustion, and the percentage of organic carbon was calculated by subtracting the carbonate content from the total soil carbon. Soil pH was measured in deionized water with a glass electrode. The soil contained 1.45% organic carbon and 0.13% total nitrogen and had a pH of 6.4. Plant material was collected near Halle (Sachsen-Anhalt) at the field site of a research network of the German Federal Ministry for Education and Research. Residues of shoots (mixture of stems and leaves) of four field-grown cultivars of maize were collected at harvest at the end of the vegetation period in October 2002. Novelis (MON810, Monsanto, http://www.monsanto.com) and Valmont (Event 176, Syngenta Seeds, http://www.syngenta.com) were transgenic cultivars; Nobilis (corresponding to Novelis) and Prelude (corresponding to Valmont) were nontransgenic isolines. The plant material was dried at 40 °C. Stems and leaves were mixed and cut with scissors into small pieces of about 5 cm length. A portion of the material was ground for analysis and incubation experiments in vitro. The concentration of the *Bt* toxin (Cry1Ab protein) was determined with ELISA (enzyme-linked-immunosorbent assay) using the QuantiPlate Kit of EnviroLogix (http://envirologix.com). The Cry1Ab protein was present, as expected, only in transgenic plant material (Table S1). Straw from Event 176 (Valmont) had a significantly lower concentration (0.842 μg g^−1^ ± 0.278 SD) than straw from MON810 (Novelis) (3.859 μg g^−1^ ± 0.527 SD), according to Tukey's test (HSD, α = 0.05).

### Experimental design

Ecosystem properties such as land use and soil structure are known to control the ratio of CO_2_ evolution per O_2_ uptake in nonsterile soils [36]. Thus, the measurement of a short-term specific respiratory rate (microbial respiration rate per unit of microbial biomass) under standardized conditions was performed to determine changes in microbial respiration activity induced by disturbance over time. Twenty-nine glass pots of 1.0 L were used, 5 × 5 for the titration (5 × 4 cultivars and 5 × 1 control) and 4 × 5 for BIOLOG (composite samples, [1 + 2] × 5 and [3 + 4] × 5). The tolerance to Cry1Ab of the microbial community was chosen to be measured by color development in BIOLOG EcoPlates and by enumeration of CFUs on agar plates.

### Soil incubation

**Figure 5:**
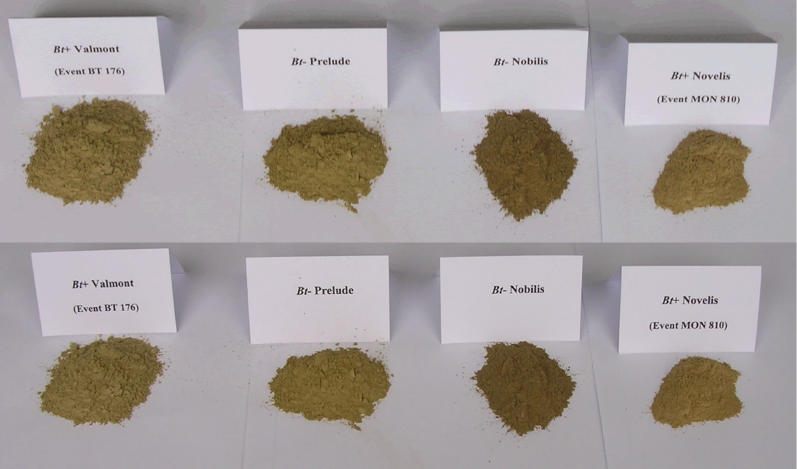
Examples of Amended Soils at the Beginning of the Experiment Some soil samples before entering the 1.0-L pots after the addition of crop residues (reaped maize straw, see Figure 1B). Please note the slightly different colors.

The incubation experiments were done in climate chambers adjusted to constant 15 °C. Two grams of ground plant residues (dry weight) were added to 50 g of sieved (2 mm) fresh soil adjusted to 75% of the water-holding capacity in glass pots/glass jars, and mixed (some soil samples after treatment are shown in Figure 5). Additionally, a control receiving the same treatment except for the application of plant material was included in the experimental design. The soils in the pots were wetted daily. CO_2_ was trapped during a complete sampling period in NaOH (0.5M and 0.2M) and detected by titration using a Metrohm 665 Dosimat according to the Isermeyer technique [37]. Respiration intervals were adjusted depending on soil activity. The detection of trapped CO_2_ and the calculation of respiration rates were performed initially over short periods (daily) and later at 3-d (twice) and 5-d (twice) intervals.

### Bacteria incubation

Bacteria were extracted from soil immediately before each titration: 15 g of soil sample, based on dry weight, was blended with 150 ml sterile buffer (10 mM bis[2-Hydroxyethyl]imino-tris[hydroxymethyl]methane (BisTris, Sigma, http://sigmaaldrich.com) at pH = 7.0) for 1 min at maximum speed and then centrifuged for 10 min at 500 *g*. According to the method of Van Beelen et al. [38], the supernatant was transferred to 2-ml sterile Eppendorf vials, rapidly frozen in liquid nitrogen, and stored at −70 °C until analysis of the community level physiological profiles (CLPP). This final freezing step differs from the original method of Rutgers et al. [39], but the CLPP of replicates made at different time points during one year were not significantly different from each other (*p* = 0.12), as most fresh suspensions of soil bacteria had very similar BIOLOG profiles and CFU plate counts compared with frozen suspensions [40,41].

### Plate counts

The colonies of viable cells were grown on 0.1TSA (Trypton Soya nutrient broth by Oxoid, http://www.oxoid.com, and agar) 5-d-old plates according to the spread plate method. To obtain the appropriate colony numbers, a series of four dilutions was made, namely 3^−8^, 3^−10^, 3^−12^, and 3^−14^. The number of CFUs was recorded after 8 d of incubation at 25 °C. This highly sensitive counting gives the best information on the density of viable cells.

### CLPP

For the analysis of the CLPP, the inoculum-density independent approach was applied [39–43]. Series of 3-fold dilutions of the bacterial suspension were produced (3^−1^ – 3^−12^), providing a complete range of color formation levels in multiwell plates of BIOLOG (http://www.biolog.com). These plates are marketed as EcoPlate [44,45], the version of BIOLOG MicroPlate specifically designed for microecological studies (the original MicroPlate was described in [42]). Aside from one water well, EcoPlates contain three replicate sets of 31 carbon sources in addition to a nutrient and salt solution and a redox dye. The plates are inoculated with 100 μl suspension per well and diluted (in 1/4 strength Ringer solution) to obtain a cell density of approximately 1 × 10^8^ cells ml^−1^ (determined by acridine orange direct counting [46]). The entire list of 31 compounds and the chemical guilds to which they belong according to [45] can be found in Table S2 and at http://www.biolog.com/pdf/eco_microplate_sell_sheet.pdf. Such sets of specific sole-carbon sources were provided specifically to test bacterial communities [40–45]. The EcoPlates were then incubated in the dark at 20 °C and at least 85% relative humidity to avoid undesired evaporation [40]. The amount of inoculum that caused 50% of the maximal theoretical response for *each* specific substrate conversion (individual WCD) was compared with the amount of inoculum that caused 50% of the maximal theoretical *average* response (AWCD) of *all* the 31 substrates in an EcoPlate (both log-transformed values), resulting in a value for the relative abundance of that specific substrate conversion [40]. Ideally the dilution series provides a complete range of color formation levels from >95% to <5% of the AWCD. Each WCD was measured individually at 590 nm during an entire week [40] using a semiautomatic sampler and a spectrophotometer (Spectra MAX250, http://moleculardevices.com). The more a specific well is colored, the more the carbon source is metabolized. Similar well patterns define, therefore, comparable carbon-source utilization.

### Statistics

Analysis of variance (PROC ANOVA) and nested (and non-nested) generalized linear models (PROC MIXED, GLM) were used to assess the effect of the concentration of the Cry1Ab protein on the microbial respiration. The strength of a relationship between each WCD and Cry1Ab value was measured by partial correlation (PROC CORR) using the sums of squares and cross-products. The fitting of multilevel models (here, the glass pots according to their treatment) and growth models (a dilution series in 12 sections of four BIOLOG EcoPlates (three sections each) comprehending 31 individual carbon sources repeated 2×) required a flexible statistical approach. The PROC MIXED routine by SAS 9.1.3 has been chosen, as PROC MIXED was written by agricultural scientists seeking a mixed generalization of linear models that allows for both fixed and random effects [47]. After invoking the procedure, three categorical variables were defined using the CLASS statement: i) addition_straw, ii) *Bt*, and iii) Cry1Ab. The MODEL statement indicates the fixed effect (each individual carbon source (thus, **A2…H4**) versus **time** as given predictor, i.e., 0, 2, 3, 7, and 21 d), and the RANDOM statement with the three nested SUB= options specifies the hierarchical multilevel structure. Obviously, additional predictors can be included in this model, but for simplicity the statement was restricted to one single **time** predictor other than the intercept. For instance, the syntax for L-arginine (**A4**) using the “between/within” (**bw**) method to compute the Denominator Degrees of Freedom (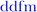) is:


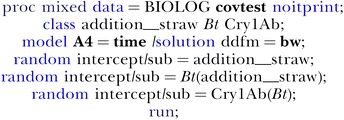


Two structural specifications in the PROC MIXED statement are the covtest option (hypothesis tests for the variance and covariance components; see [48]) and the noitprint option (to avoid the complete iteration history). The components: addition_straw (0 = no straw, 1 = added crop residues) and Bt(addition_straw), with either 0 = conventional or 1 = transgenic, are binary subject effects (dummies), whereas Cry1Ab(Bt) is a three-level (0, 0.8, 3.9 μg/g) subject effect. If the soil is amended, the class-1 unit (addition_straw = 1) is divided into one class-2 unit (Bt) with either a non-Bt or a Bt level. If transgenic (Bt = 1), this class-2 unit is further divided into one class-3 unit with three levels (high Cry1Ab, low Cry1Ab, or none at all). For example, the dilution series of bulk soil with Novelis straw inoculated into BIOLOG EcoPlates would be coded as [1 1 3.9], the conventional Prelude would be coded as [1 0 0], and the control (one bulk soil without any crop residues) would be coded as [0 0 0]. In that way four series (Nobilis, Novelis, Prelude, and Valmont) were realized for the assessment of the rate of color change in each well at d 2, 3, 7, and 21 (4×) in two replicas each, together with 4 × 2 controls at d 0 (4 × 4 × 2 + 4 × 2 = 40 observations read and used). In the running PROC MIXED model, the dimensions per subject are thus always 40 observations (of whom 32 objects are with state 1, i.e., addition_straw = 1).

The statistics of the goodness-of-fit of multiple models with the same fixed effects (here, time) but different random effects (here, crop residues, if any) is assessed by the Akaike's information criterion (AIC) and by the much more stringent Sawa's Bayesian information criterion (BIC). The AIC test penalizes for adding parameters to the model, wherefore the model with the smallest AIC value is chosen [49]. The AIC test computes the quantity:
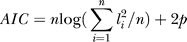
 , where the *l_i_* are the residuals from the least-squares best fit to data of the linear model,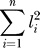 is the error sum of squares, *n* is the number of datapoints, and *p* is the number of model parameters. In a similar way, the model with the smallest Sawa's BIC value is chosen [50]. The BIC test computes the quantity:

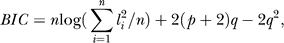
where

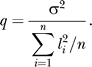
The final Type III test of fixed effects provides the significance of the chosen model (Table 3: *p*-value, and F-ratio in the text). Since effects involving class inputs consist of multiple parameters, tests of these effects have multiple degrees of freedom (here, 38 degrees of freedom). A multivariate response to time was used to assess the effect of the Cry1Ab protein on the microbial respiration, and was performed in CANOCO 4.5 [24].

## Supporting Information

Table S1 Results of the Chemical Analysis of the Maize Residues before Addition to the Microcosm (Percent of Dry Weight) The isolines of the transgenic cultivars Novelis (Event MON810) and Valmont (Event 176) and conventional cultivars Nobilis and Prelude are in roman numbers on the upper row. Valmont showed the highest contents of proteins, fats, and sugars. Using the control as appropriate nesting specification to fit the data on soil respiration to a multilevel generalized linear model (untreated or treated soils; if treated: conventional or transgenic straw; if transgenic: straw with either a low or a high Cry1Ab content), sugars became the most significant predictor for the rate of CO_2_ production (*p* = 0.0015), followed by the Cry1Ab protein (*p* = 0.0037), other proteins (*p* = 0.0134), and fats (*p* = 0.0144).
(42 KB DOC)

Table S2 List of the BIOLOG EcoPlate Carbon Sources with the Pearson Product-Moment Correlation Coefficient, *r,* between the Specific Sources and the Concentration of the Cry1Ab Protein as Measured at the Beginning of the Experiment (Table S1) (41 KB PDF)

## Abbreviations

AIC: Akaike's information criterion
BIC: Bayesian information criterion
CFU: colony-forming unit
CLPP: community level physiological profiles
WCD: well-color development
