# Supplementary material for: Can Transgenic Maize Affect Soil Microbial Communities?
Source: PLoS Comput Biol. 2006 Sep 29;2(9):e128. doi: 10.1371/journal.pcbi.0020128 (PMC1584322; doi:10.1371/journal.pcbi.0020128)
Supplement: Table S1 — The isolines of the transgenic cultivars Novelis (Event MON810) and Valmont (Event 176) and conventional cultivars Nobilis and Prelude are in roman numbers on the upper row. Valmont showed the highest contents of proteins, fats, and sugars. Using the control as appropriate nesting specification to fit the data on soil respiration to a multilevel generalized linear model (untreated or treated soils; if treated: conventional or transgenic straw; if transgenic: straw with either a low or a high Cry1Ab content), sugars became the most significant predictor for the rate of CO2 production (p = 0.0015), followed by the Cry1Ab protein (p = 0.0037), other proteins (p = 0.0134), and fats (p = 0.0144). (42 KB DOC) [file pcbi.0020128.st001.doc]

**Table S1**

**Supplementary Information for *Transgenic Maize Affects Soil Bacteria* by:**

**Christian Mulder, Marja Wouterse, Markus Raubuch, Willem Roelofs, Michiel Rutgers**

**Table S1.** Results of the chemical analysis of the maize residues before addition to the microcosm (% of dry weight). The isolines of the transgenic cultivars ‘Novelis’ and ‘Valmont’ and conventional cultivars ‘Nobilis’ and ‘Prelude’ are in Roman numbers on the upper row. ‘Valmont’ showed the highest contents of proteins, fats and sugars. Using the control as appropriate nesting specification to fit our data on soil respiration to a multilevel Generalized Linear Model (untreated or treated soils; if treated: conventional or transgenic straw; if transgenic: straw with either a low or a high Cry1Ab content), sugars became the most significant predictor for the rate of CO_2_ production (*p* = 0.0015), followed by the Cry1Ab protein (*p* = 0.0037), other proteins (*p* = 0.0134) and fats (*p* = 0.0144).

|  | **I+** | **II+** | **I** | **II** |
| --- | --- | --- | --- | --- |
|  | ***Novelis***  **(MON 810)** | ***Valmont***  **(Event 176)** | ***Nobilis* (conventional)** | ***Prelude* (conventional)** |
|  |  |  |  |  |
|  |  |  |  |  |
| Microbial C : N ratio | 24.13 | 32.83 | 17.20 | 18.73 |
| Organic carbon (%) | 42.9 | 42.8 | 42.0 | 44.0 |
|  |  |  |  |  |
| Proteins (%) | 4.31 | 5.36 | 4.50 | 4.82 |
| Fats (%) | 0.87 | 1.02 | 0.88 | 0.89 |
| Sugars (%) | 0.85 | 1.11 | 0.68 | 0.60 |
| Starch (%) | 0.12 | 0.29 | 0.22 | 0 |
|  |  |  |  |  |
| Lignin (%) | 6.49 | 6.09 | 5.84 | 7.11 |
| Hemicellulose (%) | 28.84 | 24.45 | 30.82 | 24.03 |
| Cellulose (%) | 41.89 | 42.58 | 42.20 | 41.17 |
|  |  |  |  |  |
|  |  |  |  |  |
| Cry1Ab (ng g^-1^) | 3859 | 842 | 0 | 0 |
|  |  |  |  |  |

Found at DOI: 10.1371/journal.pcbi.****
