# Supplementary material for: Can Transgenic Maize Affect Soil Microbial Communities?
Source: PLoS Comput Biol. 2006 Sep 29;2(9):e128. doi: 10.1371/journal.pcbi.0020128 (PMC1584322; doi:10.1371/journal.pcbi.0020128)
Supplement: Table S2 — (41 KB PDF) [file pcbi.0020128.st002.pdf]

Table S2

Supporting Information for *Transgenic Maize Affects Soil Bacteria*

by: Christian Mulder, Marja Wouterse, Markus Raubuch, Willem Roelofs, Michiel Rutgers

Table S2. List of the BIOLOG carbon sources (Figure 2) with the Pearson product-moment correlation coefficient, r, between the specific sources and the concentrations of the Cry1Ab protein as measured at the beginning of the experiment (Table S1). Found at DOI: 10.1371/journal.pcbi.****

BIOLOG EcoPlate C-sources		*r*	*P*	
Water (control)	A1	_	_	
β-methyl-D-glucoside	A2	0.349	**0.027**	Carbohydrate
D-galactonic acid γ-lactone	A3	0.177	0.274	Carbohydrate
L-arginine	A4	0.016	0.923	Amino acid
Pyruvic acid methyl ester	B1	-0.322	**0.043**	Carboxylic acid
D-xylose	B2	0.390	**0.013**	Carbohydrate
D-galacturonic acid	B3	0.204	0.208	Carboxylic acid
L-asparagine	B4	0.160	0.324	Amino acid
Tween 40	C1	-0.183	0.258	Polymer
i-erythritol	C2	-0.347	**0.028**	Carbohydrate
2-hydroxy benzoic acid	C3	-0.097	0.551	Phenolic compound
L-phenylalanine	C4	-0.188	0.246	Amino acid
Tween 80	D1	-0.273	0.088	Polymer
D-mannitol	D2	0.348	**0.028**	Carbohydrate
4-hydroxy benzoic acid	D3	-0.410	**0.009**	Phenolic compound
L-serine	D4	0.269	0.093	Amino acid
α-cyclodextrin	E1	-0.372	**0.018**	Polymer
N-acetyl-D-glucosamine	E2	0.679	**<.0001**	Carbohydrate
γ-hydroxybutyric acid	E3	-0.196	0.226	Carboxylic acid
L-threonine	E4	0.162	0.318	Amino acid
Glycogen	F1	0.331	**0.037**	Polymer
D-glucosaminic acid	F2	-0.229	0.156	Carboxylic acid
Itaconic acid	F3	-0.182	0.260	Carboxylic acid
Glycyl-L-glutamic acid	F4	-0.310	0.051	Amino acid
D-cellobiose	G1	0.459	**0.003**	Carbohydrate
Glucose-1-phosphate	G2	0.464	**0.003**	Carbohydrate
α-ketobutyric acid	G3	0.027	0.869	Carboxylic acid
Phenylethylamine	G4	-0.187	0.247	Amine
α-D-lactose	H1	0.371	**0.019**	Carbohydrate
D,l-α-glycerol phosphate	H2	0.253	0.116	Carbohydrate
D-malic acid	H3	0.252	0.117	Carboxylic acid
Putrescine	H4	-0.040	0.806	Amine
